# Interspecific Competition between the House Fly, *Musca domestica* L. (Diptera: Muscidae) and Black Soldier Fly, *Hermetia illucens* (L.) (Diptera: Stratiomyidae) When Reared on Poultry Manure

**DOI:** 10.3390/insects10120440

**Published:** 2019-12-07

**Authors:** Chelsea D. Miranda, Jonathan A. Cammack, Jeffery K. Tomberlin

**Affiliations:** Department of Entomology, Texas A&M University, 2475 TAMU, College Station, TX 77845, USAjktomberlin@tamu.edu (J.K.T.)

**Keywords:** mass-production, priority effect, biological control

## Abstract

Few studies have examined the competitive interaction between the house fly (HF) and the black soldier fly (BSF). The fact that the BSF deters HF oviposition is widely cited in BSF literature, but this interaction has not been assessed in over three decades. In this study, the competitive interaction of BSF and HF larvae was observed on fresh (day 0) and aged poultry manure (manure aged for two, four, six, or eight days). Specifically, a priority effect study was conducted to determine if colonization sequence influences time to first pupariation (HF) or pre-pupation (BSF), survivorship, and weight. Results show >70% of HFs reached pupariation in all treatments except when placed on manure eight days after the initial inoculation with BSF. However, age of the resource negatively impacted time to first pupariation and puparium weight when HFs were reared alone or introduced two to eight days after BSF. No BSF pre-pupae resulted from treatments in which HFs were the pioneering species. BSFs reached the highest percent pre-pupation when reared alone on fresh manure, but BSFs may be more susceptible to the negative impacts of an aging resource, as no pre-pupae were observed when provided with six- or eight-day-old manure. Similar to HFs, age of the resource may have impacted development and survivorship; other factors such as moisture content, chemical composition, and amount of resource provided may have also impacted our results. These data may be useful in implementing BSFs as biological control agents of the HF, as well provide valuable information for facilities mass-producing HFs or BSFs for food or feed.

## 1. Introduction

Competition is an interaction that occurs between individuals (of the same or different species) sharing a common resource, potentially resulting in reduced growth, reproduction, and/or survivorship [1]. Understanding how competition operates in nature or mass-rearing environments is important, as it may assist in reducing pest populations. In agriculture, pests of livestock can cause substantial economic loss [2]. Some of the arthropods associated with animal production rely on manure as a resource, and as a result, manure is often the site of intense competition. Two species that use manure as a resource for offspring development are the house fly (HF), *Musca domestica* L. (Diptera: Muscidae) and black soldier fly (BSF), *Hermetia illucens* (L.) (Diptera: Stratiomyidae).

House flies are known pests that can develop to pupariation on bovine, swine, or poultry manure in as little as five to eight days [3,4]. They are vectors of numerous pathogens and may cause economic loss in animal production systems [5] via costs associated with fly control and could lead to contentious interactions between farmers and the public [6]. For these reasons, much of the research conducted on HFs focuses on control efforts with pesticides [7,8,9]. Unfortunately, due to decades of pesticide use, HFs have become resistant to many compounds [10,11,12]. Therefore, control of HFs should involve an integrated approach, combining cultural, biological, and chemical methods [13]. Of the available options for biological control, using BSFs is an attractive alternative; however, little is known about what governs HF control by BSFs.

The BSF is found in temperate and tropical regions throughout the world. Historically, it was considered a pest [14], but due to its ability to reduce a variety of wastes [15] and convert them into valuable biomass of approximately 42% protein and 35% fat [16], reduce dry matter and nutrients by 50% or more [17,18] and reduce odorous volatile compounds [19], this species is now considered beneficial. Furthermore, BSFs also inhibit house fly oviposition [17,20,21,22]. Thus, BSFs offer a means to control HFs, which may ultimately reduce pesticide dependence. The mechanism underlying how BSFs deter HFs is unknown but appears to be related to changes in the microbial communities within the substrate. For example, BSFs are known to reduce *Escherichia coli* [23,24], which HFs utilize for growth and development [25,26]. Despite the previous suppositions, the competitive interaction between HFs and BSFs has not been assessed in over three decades and additional work is needed to better understand the factors that govern this interaction.

Rising interest in the production of BSFs for feed, coupled with the fact that HFs are often pests in such facilities, calls for further investigation of their relationship. This study was conducted to gain a better understanding of the competitive interactions between the larvae of HFs and BSFs. Specifically, we aimed to determine if colonization sequence influenced development time to pupariation (HF) or pre-pupation (BSF), survivorship, and weight. This will provide insight on the mechanisms governing HF control when BSFs are present and may be valuable for on-farm control of HFs, or for industrial applications of BSFs where eliminating pest persistence is the goal.

## 2. Materials and Methods

Methods for this experiment in regard to the colonies used, collection of manure, and colony maintenance are based on those conducted by Miranda, Cammack and Tomberlin [4,27] House fly larvae were obtained from a colony at the Forensic Laboratory for Investigative Entomological Sciences (F.L.I.E.S.) Facility at Texas A&M University, College Station, TX, USA, which was maintained by providing water, as well as a mixture of sugar and non-fat milk powder (Great Value^®^ Brand, Wal-Mart^®^ Stores, Inc., Bentonville, AR, USA) (1:1 ratio by mass) to adults *ad libitum*. The colony originated in 2014 from adults collected from dairy and swine facilities in Stephenville, TX, USA. Black soldier fly larvae in this experiment were from a colony established in 2014 maintained at the F.L.I.E.S. Facility at Texas A&M University, College Station, TX, USA using a modified version of the methods detailed by Sheppard et al. [28]. This colony originated from a colony at the Coastal Plains Experiment Station (University of Georgia) in Tifton, GA, USA.

Poultry manure less than 12 h old was collected from layer hens at the Poultry Science Research, Teaching, and Extension Center (Texas A&M University) located in College Station, TX, USA. Manure was placed into 18.9 L buckets with lids (Home Depot^®^, Leaktite^TM^, Leominster, MA, USA) and transported to the F.L.I.E.S Facility, where it was mixed vigorously by hand for approximately 5 min and transferred to 3.76 L Ziploc^®^ Freezer Bags (S.C. Johnson & Son, Racine, WI, USA) and stored at −20°C until used. Manure was allowed to thaw at room temperature for 24 h before initiation of the experiment and manure that was not used to initiate the experiment was placed in a 1.9 L Reditainer™ EXTREME FREEZE™ deli container with a lid (Clear Lake Enterprises, Port Richey, FL, USA) and stored in a refrigerator (4°C) until used. The initial moisture content of the manure was 76% and was determined gravimetrically with three 10 g samples of thawed manure [29].

To collect HF eggs, a Kimwipe^®^ (Kimberly-Clark Corp., Irving, TX, USA) saturated with evaporated milk (Great Value^®^ Brand, Wal-Mart^®^ Stores, Inc., Bentonville, AR, USA) was balled up and placed in an 88 mL bathroom cup (Great Value^®^ Brand, Wal-Mart^®^ Stores, Inc., Bentonville, AR, USA). The cup was placed inside a 30 cm × 30 cm × 30 cm cage (BioQuip^®^ Products, Rancho Dominquez, CA, USA) with gravid adult flies and checked for egg deposition every four hours. The Kimwipe^®^ was unfolded and rinsed with distilled water over a 100 mL beaker to collect viable eggs that sank to the bottom of the beaker. Eggs were collected with a glass Pasteur pipette and transferred to a damp Kimwipe^®^ that was placed inside an 88 mL bathroom cup (Great Value^®^ Brand, Wal-Mart^®^ Stores, Inc., Bentonville, AR, USA) covered with a Kimwipe^®^ held in place with a rubber band. The cup was stored in a Rheem Environmental Chamber (Asheville, NC, USA; at 26.00 ± 0.04°C, 63.00 ± 8.72% relative humidity (RH), and 16 h light/8 h dark) until larval eclosion.

Black soldier fly adults were maintained in a 1.2 m × 1.2 m × 2.4 m cage constructed of wood and fiberglass window screening (18 × 16 mesh size) sides in a greenhouse (25–30°C, >50% RH). Corrugated cardboard was cut into 4.0 cm × 4.0 cm × 0.5 cm pieces and three pieces of cardboard were taped together to form a bundle, which was placed on the lid of a 5.7 L Sterilite^®^ container with a 15 cm × 7 cm hole covered with fiberglass window screen. Approximately 500 g Gainesville diet (50% wheat bran, 30% alfalfa meal, 20% corn meal) [30] saturated with reverse osmosis water was placed inside the Sterilite^®^ container and served as an attraction medium. The cardboard was checked for egg clutches every four hours, after which the cardboard was dissected, and the egg clutches removed. The eggs were placed in a 0.5 L plastic container, covered with a paper towel held in place with a rubber band, and stored in the Rheem Environmental Chamber described above until larval eclosion.

**Figure 1:**
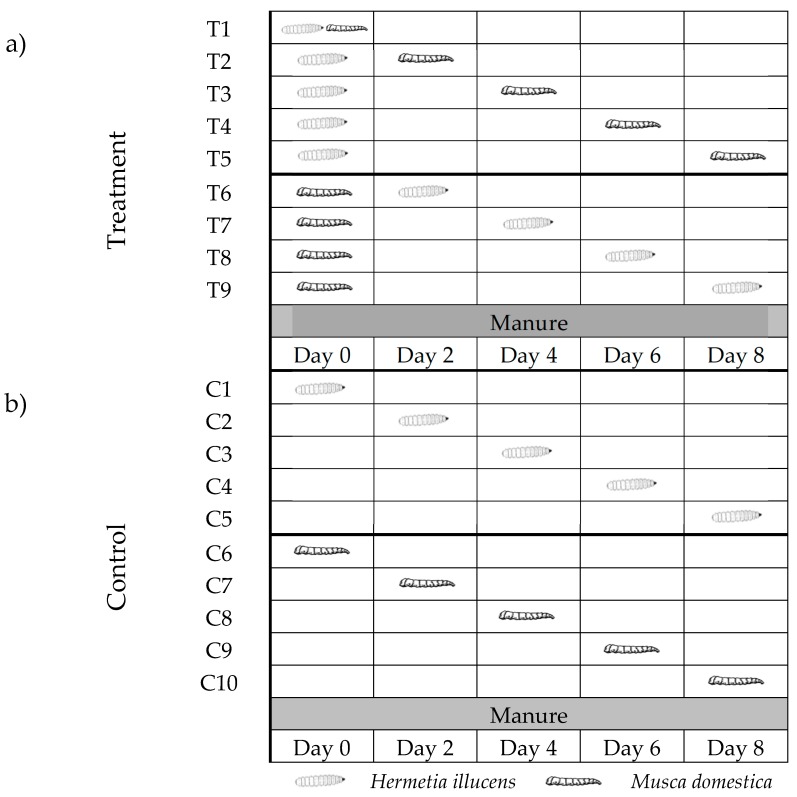
Experiment design of black soldier fly (BSF) and house fly (HF) larvae fed poultry manure aged for 0–8 d at 26°C, 70% relative humidity (RH), 16 h light:8 h dark.(**a**) In mixed treatments, the pioneer species, BSF, was placed on fresh manure, while the competing species, HF, was placed simultaneously (T1) or 2 days, (T2), 4 days (T3), 6 days (T4) or 8 days (T5) after the pioneering species. The study was reversed so that HF represented the pioneering species (T6–T9) and BSF were introduced 2 days, 4 days, 6 days, or 8 days after the initial introduction of HF. (**b**) Pure (single species) cultures of larvae from each species (BSF and HF) were placed on fresh (C1, C6), 2-day-old (C2, C7), 4-day-old (C3, C8), 6-day-old (C4, C9) or 8-day-old (C5, C10) manure. This experiment design is based on the design by Brundage, Benbow and Tomberlin [31].

Replicates consisted of 60 g of poultry manure placed inside 0.5 L deli containers. Manure placed inside the cups was inoculated with 100 newly-hatched (<12 h old) first instar house flies and introduced into cups on day 0, 2, 4, 6 and 8 after the introduction of 100 newly-hatched (<12 h old) first instar BSF (Figure 1; T1–T5). Treatments with BSF as the pioneering species are referred to hereafter as BSF0HF2, BSF0HF4, BSF0HF6, and BSF0HF8. For example, BSF0HF2 indicates BSF were placed on the manure on day 0 and HF were introduced on day 2. The introduction times were selected because they represent the beginning (0 d), middle (2 and 4 d), and end (6 and 8 d) of HF development on poultry manure [31]. The study was reversed so that house flies represented the pioneering species and first instar BSF were inoculated into the manure on day 0, 2, 4, 6 and 8 after the introduction of first instar HF (Figure 1; T6–T9). These treatments are referred to as HF0BSF2, HF0BSF4, HF0BSF6, and HF0BSF8. To ensure that results were not due to resource age, 100 newly hatched larvae of each species were placed on manure aged for 0, 2, 4, 6 or 8 d without testing priority effects, and served as controls (Figure 1; C1–C10). These treatments are referred as HF0, HF2, HF4, HF6, and HF8 for house flies and BSF0, BSF2, BSF4, BSF6, and BSF8 for black soldier flies. Each treatment and control were replicated three times per trial and two trials were conducted. Cups with larvae were placed in the Rheem Environmental Chamber and maintained at the conditions described above. Cups were monitored daily for puparium (HF) or pre-pupae (BSF). Puparium and pre-pupae were removed daily from the cups, weighed on an OHaus^®^ Adventurer Pro AV64 balance (OHaus^®^ Corporation, Pine Brook, NJ, USA). Weight, percent pupariation (HF) or pre-pupation (BSF), as well as time (days) to first pupariation (HF) or pre-pupation (BSF) were recorded. The terms puparium and pupariation are used to describe the metamorphosis of the final larval instar of cyclorrhaphous flies, such as HFs. Pupariation is the formation of the puparium, or the barrel-like structure formed via muscle contractions and modifications of the cuticle of the last larval instar [32,33]. This phase is different from pupation, which occurs hours after pupariation and is the is the formation of the pupa inside the puparium [32,33]. It is impossible to determine when pupation occurs unless the puparium is dissected [32,33], which was not performed during this study; therefore, the term puparium (instead of pupa/pupal) is used throughout the text. The final larval instar of the BSF is known as the pre-pupa and can be distinguished from earlier larval instars by the change in the color of the cuticle from white to dark brown [34]. These stadia were selected because they signify the initiation of intrapuparial development [33].

Time (days) to first pupariation (HF) and first pre-pupation (BSF), percent pupariation (HF), percent pre-pupation (BSF), puparium (HF) and pre-pupal (BSF) weight across treatments were analyzed. An ANOVA was performed for each of the parameters listed above using JMP 14.0.0 (SAS Institute Inc., Cary, NC, USA). Tukey’s honest significant difference (HSD) was used for mean separation (*p* ≤ 0.05).

## 3. Results

### 3.1. Time to First Pupariation (HF)

**Figure 2:**
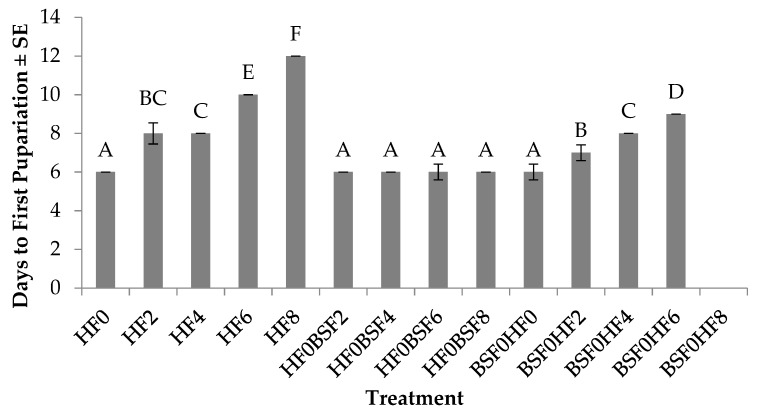
Days to first pupariation (mean ± standard error (SE), ^1^n = 6) for house fly (HF) larvae reared on poultry manure (aged for 0–8 d), alone, or with black soldier fly (BSF) larvae, at 26°C, 70% RH, 16 h light:8 h dark. Different letters indicate significant differences between treatments, ANOVA followed by Tukey’s honest significant difference (HSD) (α = 0.05). ^1^n = number of replicates per treatment.

Time (days) to first pupariation for HFs was significantly different (F_12, 52_ = 326.8; *p* < 0.0001) across treatments. No significant treatment by trial interaction (F_12, 52_ = 0.6000; *p* = 0.8321) or trial effect were determined (F_1, 52_ = 3.20; *p* = 0.0795). In regard to larvae in the control groups, HFs placed on manure on day 0 developed the fastest (six days), whereas those placed on manure aged for two to eight days took 1.5–10 days longer to develop (Figure 2). For those placed on manure in which HFs were the pioneering species and BSF were introduced at day 0–8, larvae in all treatments developed to pupariation in approximately six days. In treatments where BSFs were the pioneering species, the shortest development time (six days) was found in treatments where HFs were introduced on fresh manure, and this was one to three days faster than treatments where HF were introduced in manure aged for two to six days. House fly larvae placed on manure eight days after the initial introduction of BSF did not reach pupariation.

### 3.2. Percent Pupariation (HF)

**Figure 3:**
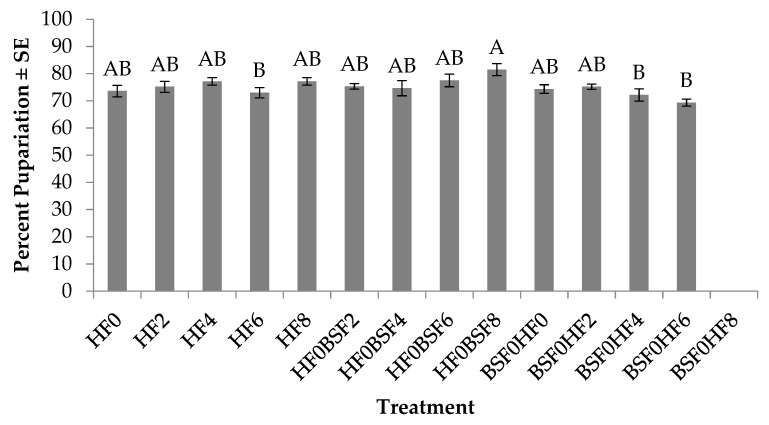
Percent pupariation (mean ± SE, ^1^n = 6) for house flies (HF) when reared on poultry manure (aged 0–8 d), alone, or with black soldier fly (BSF) larvae, at 26°C, 70% RH, 16 h light:8 h dark. Different letters indicate significant differences between treatments, ANOVA followed by Tukey’s HSD (α = 0.05). ^1^n = number of replicates per treatment.

Percent pupariation for HFs differed significantly across treatments (F_12, 52_ = 2.9; *p* = 0.0033). No treatment by trial interaction was found (*p* = 0.7069); however, a significant trial effect (F_1, 52_ = 16.6; *p* = 0.0002) was. In general, 4% more individuals reached pupariation in trial one than in trial two. The highest percent pupariation in the control groups was when HFs were placed on manure aged for four days (77%), which was up to 4% more than those placed on either fresh manure or manure aged for two days, six days or eight days (Figure 3). In the mixed treatments in which HFs were the pioneering species, the highest percent pupariation was found in treatments where BSFs were introduced on eight-day-old manure (81%). This was 4–6% more than treatments in which BSF were placed on manure aged for two days, four days or six days. For treatments with BSFs as the pioneering species, the highest percent pupariation was found in treatments in which HFs were introduced on manure after two days (75%), and this was 1–6% more than those that had HF introduced on fresh manure, or manure aged for four or six days, and 7% more than those placed on manure with BSFs at the same time. No HF larvae placed on manure eight days after the initial introduction of BSFs were found to reach pupariation.

### 3.3. Puparium Weight (HF)

**Figure 4:**
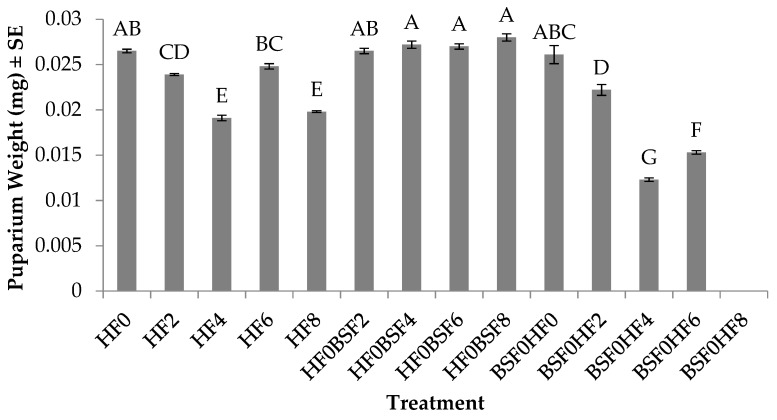
Puparium weights (mg) (mean ± SE, ^1^n = 6) of house flies (HF) when reared on poultry manure (aged 0–8 d), alone, or with black soldier fly (BSF) larvae, at 26°C, 70% RH, 16 h light:8 h dark. Different letters indicate significant differences between treatments, ANOVA followed by Tukey’s HSD (α = 0.05). ^1^n = number of replicates per treatment.

Puparium weight for HFs was significantly different (F_12, 52_ = 124.8; *p* < 0.0001) across treatments. No significant treatment by trial interaction was found (*p* = 0.9993), but a significant trial effect was (F_1, 52_ = 4.72; *p* = 0.0343). In general, individuals in trial two weighed 2% more than those in trial one. In regard to larvae in the control groups, individuals produced on fresh manure were the heaviest (26 mg) and weighed 6–28% more than those placed on manure aged for two to eight days (Figure 4). For those placed in mixed treatments where HFs were the pioneering species, the heaviest weight was recorded in treatments in which BSF were placed on day 8 (28 mg), and they weighed 3–7% more than when BSF were introduced to the manure on days 0–6. In treatments where HFs were introduced with, (on day 0) or after, BSF (on day 2, 4 or 6), HFs placed on manure on day 0 weighed the most (26 mg) and this was 15–53% more than HF larvae placed on manure aged for 2–6 days. House fly larvae placed on manure eight days after the initial introduction of BSF did not survive to pupariation.

### 3.4. Time to First Pre-pupation (BSF)

**Figure 5:**
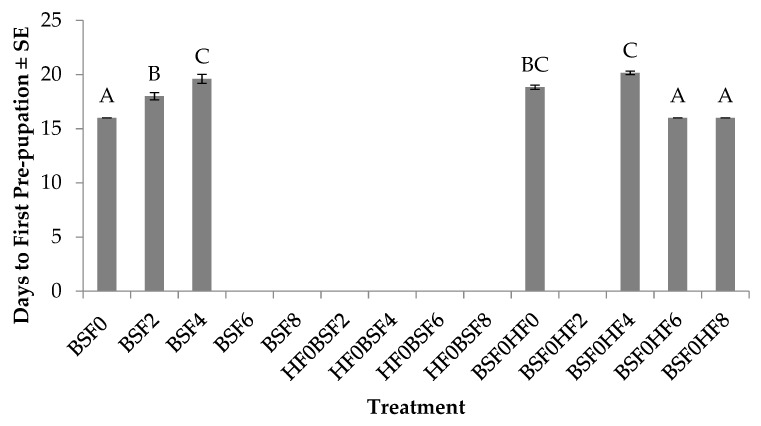
Time (days) to first pre-pupation (mean ± SE, ^1^n = 6) of black soldier flies (BSF) when reared on poultry manure (aged 0–8 d), alone, or with house flies (HF), at 26°C, 70% RH, 16 h light:8 h dark. Different letters indicate significant differences between treatments, ANOVA followed by Tukey’s HSD (α = 0.05). ^1^n = number of replicates per treatment.

Time (days) to first pre-pupation of BSFs was significantly different across treatments (F_6, 27_ = 37.9; *p* < 0.0001). No treatment by trial interaction (*p* = 0.9389) or trial effect (*p* = 0.6770) were found. In regard to larvae in the control groups, BSF placed on manure on day 0 developed the fastest (16 days), whereas those placed on manure aged for two days or four days took two to four days longer (Figure 5). No pre-pupae were found in treatments in which larvae were placed alone on manure aged for six days or eight days. Additionally, no pre-pupae were found in mixed treatments in which HFs were the pioneering species. However, in treatments in which BSFs served as the pioneering species, the fastest development (16 days) was found in treatments with HFs introduced on day 6, and this was two to four days less than those with HFs introduced at the same time as BSF, or four days after BSF.

### 3.5. Percent Pre-pupation (BSF)

**Figure 6:**
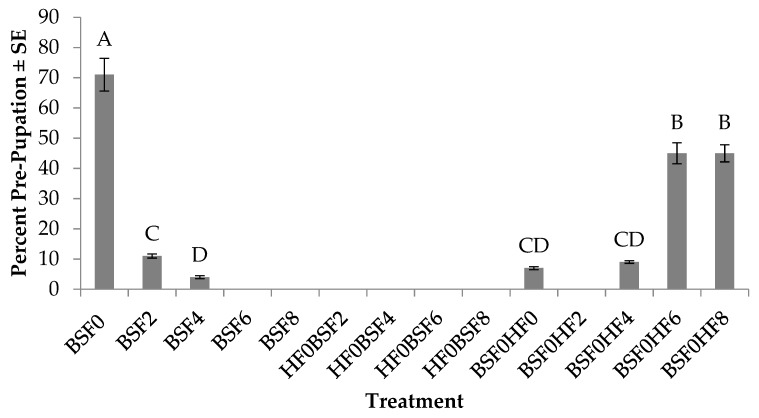
Percent pre-pupation (mean ± SE, ^1^n = 6) of black soldier flies (BSF) when reared on poultry manure (aged 0–8 d), alone, or with house flies (HF), at 26°C, 70% RH, 16 h light:8 h dark. Different letters indicate significant differences between treatments, ANOVA followed by Tukey’s HSD (α = 0.05). ^1^n = number of replicates per treatment.

Percent pre-pupation of BSFs was significantly different across treatments (F_6, 27_ = 83.3; *p* < 0.0001). No treatment by trial interaction (*p* = 0.9431) or trial effect (*p* = 0.5475) were found. In regard to larvae in the control groups, the highest percent pre-pupation (71%) was found in treatments with BSF placed on manure on day 0, and this was 60–68% more than those inoculated into manure aged for two to four days (Figure 6). In mixed treatments with BSF as the pioneering species, the highest percent pre-pupation (45%) was found in treatments with HF introduced on day 6 or day 8, and this was 34–41% more than those with HF introduced on day 0, 2, or 4.

### 3.6. Pre-pupal Weight (BSF)

**Figure 7:**
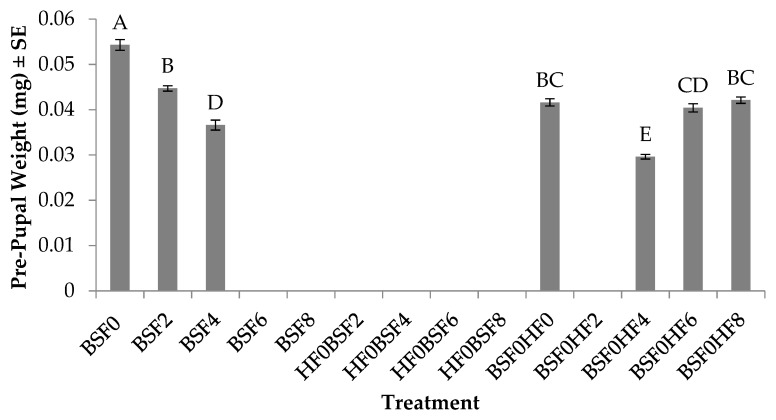
Pre-pupal weight (mg) (mean ± SE, ^1^n = 6) of black soldier flies (BSF) when reared on poultry manure (aged 0–8 d), alone, or with house flies (HFs)), at 26°C, 70% RH, 16 h light:8 h dark. Different letters indicate significant differences between treatments, ANOVA followed by Tukey’s HSD (α = 0.05). ^1^n = number of replicates per treatment.

Pre-pupal weight of BSFs was significantly different across treatments (F_6, 27_ = 72.5; *p* < 0.0001). No treatment by trial interaction (*p* = 0.9832), but a significant trial effect (F_1, 56_ = 12.6310; *p* = 0.0014) was found. In general, individuals in trial two weighed 6% more than those in trial one. In regard to larvae in the control groups, the heaviest pre-pupae (53.1 mg) were found in treatments with BSF placed on manure on day 0 and they were 19–45% more than those inoculated into two to four-day-old manure (Figure 7). In mixed treatments with BSF as the pioneering species, the heaviest pre-pupae (42.1 mg) were found in treatments where HFs were introduced on day 8, and this was 1–42% more than those where HFs were introduced on day 0, 4, or 6.

## 4. Discussion

Results from this study show that colonization sequence impacts development time, survivorship, and weight of HFs and BSFs. More specifically, HFs are able to successfully develop to pupariation as the pioneer species, before any obvious negative impact due to the presence of the later colonizer, BSFs, is apparent (Figure 2). In general, HFs successfully reached >70% pupariation in all but one treatment (BSF0HF8) regardless of whether they were placed on manure alone (control) or introduced before or after BSFs (mixed treatments) (Figure 3). A likely explanation for the poor performance of HFs in BSF0HF8 may be due to age of the resource, as HF development time was extended by two to six days when reared alone on eight day old manure (Figure 2) and puparium weight was reduced by up to 25% (Figure 4) when compared to those placed on manure at earlier times. The age of the manure, combined with the fact that BSFs likely exhausted the majority of the nutrients and the moisture available in eight days, may explain why HFs performed poorly when introduced eight days after BSFs.

In regard to BSF performance, there are obvious negative impacts on BSF growth and survivorship, which may be due to the presence of HFs, but may also be due to age of the resource or amount of resource provided during the experiment. Out of 14 treatments with BSFs, pre-pupae were found in only seven treatments. Of those seven, three of them were controls (BSF0, BSF2, and BSF4) in which development time was extended by up to three days (Figure 5), and 4–71% survived on fresh manure or manure aged for two to four days (Figure 6). Similar to the HF results, the age of the resource likely impacted BSF development as no BSF pre-pupae were found in treatments with manure aged for six days or eight days (BSF6 or BSF8). In mixed treatments in which HFs were the pioneering species (HF0BSF2, HF0BSF4, HF0BSF6, and HF0BSF8), no BSF pre-pupae were found, although larvae were detected in all of the mixed treatments at the end of the experiment. However, as the pioneering species, BSF survivorship increased from 7% in treatments with HFs introduced at the same time as BSF (BSF0HF0), to 45% in treatments with later HF introductions (BSF0HF6 or BSF0HF8) (Figure 6). No BSF pre-pupae were found in treatments in which HFs were introduced after BSF on day 2 (BSF0HF2). Interestingly, BSF larvae were found in all BSF0HF2 replicates, but they did not develop to pre-pupae. The cause of this is unknown, but it is possible that BSFs were not able to meet their metabolic requirements for early larval development [35] before HFs arrived. In regard to weight, BSFs weighed 21–48% more when placed on fresh manure when compared to those placed on manure aged for two to four days (BSF2 and BSF4) (Figure 7). In mixed treatments where HFs were introduced on day 8 (BSF0HF8), BSFs weighed 1% more than those placed with HFs (BSF0HF0) and 20–42% more than those with HFs placed on manure at later times (BSF0HF4 or BSF0HF6). When comparing BSF results to those for HFs, the age of the resource impacted the development of both species, although BSFs were susceptible to an aging resource. Furthermore, HFs had a greater impact on BSF performance as early introductions inhibited BSF development. These results demonstrate that HFs are a true pest as they are able to persist in most treatments and, more importantly, their presence can be detrimental in a BSF mass-rearing facility.

Previous research has shown that colonization sequence may govern fitness among competitors. By definition, this is known as a priority effect, which has been observed in blow flies (Diptera: Calliphoridae) [36], mosquitoes (Diptera: Muscidae) [37], beetles (Coleoptera: Cerambycidae, Scolytidae, and Trogossitidae) [38], and ants (Hymenoptera: Formicidae) [39]. However, few studies have examined the competitive interaction between HFs and BSFs, and none have incorporated colonization sequence as a factor. Initial research efforts on HFs and BSFs focused on how the presence or absence of BSFs influenced abundance of HFs. Furman, Young and Catts [20] noted there was an absence of HFs when BSFs were present at poultry facilities. This observation led to an experiment in which gravid HF females were exposed to two containers: one with manure and 500 BSF larvae, and the other with only manure. The researchers found that HFs emerged from the container with manure only, but no HFs were observed from the container with BSFs. This study went further to suggest a density-dependent response for HFs, such that as BSF larval density increased, percent emergence and survivorship for all HF life stages decreased. In a similar study, Bradley and Sheppard [21] showed that BSF density as well as the amount of time that BSFs occupy the resource negatively impacts oviposition by HFs. Other studies showed that control of BSFs with larvicides [14] or by cultural control (i.e., cleaning manure basins) [17] reduces BSF populations and subsequently increases HF populations. Yet despite all of these findings, facilities that mass-produce BSFs are still threatened by HF infestations. As such, it is possible that colonization sequence is a factor that governs successful HF infestations if HFs are able to colonize the resource before the resource ages or BSFs reduce the moisture or microbes.

Development and survivorship of both species were impacted by the age of the resource. Although not tested in this study, it is possible that variations in the moisture content of aged manure may explain why no BSF pre-pupae were found in treatments with HFs as the pioneering species (HF0BSF2, HF0BSF4, HF0BSF6, and HF0BSF8). As larvae consume the resource, they likely mechanically aerate it via contractions of their body [40], thereby reducing the moisture content. Past research has shown that BSFs cannot develop on resources with low moisture contents [41], and although the lower threshold is not known, it likely is between 40–55% moisture [42]. Fatchurochim, Geden and Axtell [41] found similar results for BSFs and HFs, and this may also explain why no HF pupae were found in BSF0HF8 treatments. However, moisture may not be the only explanation for variation in our results, as the nutrient content of manure changes with age, which also could have impacted development.

The chemical composition of manure changes with age. Microbes break down nutrients in manure, and over time, these nutrients are volatilized as secondary metabolites [43]. Although HF development time and weight were reduced when fed aged manure, pupae were found in all controls, suggesting there were sufficient nutrients available to support growth on an aging resource. However, no HF pupae were found in the mixed treatment BSF0HF8, and this may be due to depletion of nutrients, or the impact of BSFs on bacteria [44]. Black soldier flies are known to reduce *E*. *coli* in poultry manure in as little as three days [23], and house flies depend on *E*. *coli* to obtain essential nutrients for proper growth and development [25]. Although not tested in this study, perhaps BSFs reduced the nutrient content as well as crucial bacteria, such as *E*. *coli*, before HFs were introduced, and thus limited HF performance in these treatments. From an industrial standpoint, this insight is valuable in controlling HF infestations, as overfeeding BSFs may hinder their ability to reduce the microbes essential for HF development [24] and could result in frequent pest infestations. Consequently, optimizing feed rates may be influential in HF control, but more importantly, the amount of resource provided may also be responsible for poor BSF performance in this experiment.

The amount of food provided may have impacted BSF performance. Black soldier flies are larger individuals with longer development time to the pre-pupal stage (16–20 days), and consequently require more resources than HFs [45]. Larval nutrition is critical to BSFs, as they are acquiring all of the nutrients needed to sustain adult livelihood. House flies differ from BSFs in that they require less resources because of their short development time to pupariation (six days) (Figure 2). They may also invest less in nutrient acquisition during larval development because they have fewer digestive enzymes compared to BSFs [46], or because they must feed as adults to mature reproductively [47]. Black soldier flies, on the other hand, do not necessarily have to feed as adults [28] although they can [48,49]; there is an adaptive value for BSFs to invest in more digestive enzymes to feed more efficiently as larvae and acquire all of the nutrients needed to sustain adult livelihood [50].

The amount of food provided was determined by preliminary experiments that resulted in low survivorship (<20%) when newly hatched BSF larvae were placed on ≥100 g of poultry manure. It appeared that the young larvae were not able to process the manure before unfavorable microbes proliferated [51,52]; therefore, in an effort to avoid overfeeding and reduce the effect of competition, the minimum amount of fresh poultry manure for BSFs was determined to be 0.6 g/larva (>70% survivorship). However, as previously discussed, moisture and nutrient content are reduced in aging manure, and manure with HFs. The amount of manure selected for this experiment may not have been an adequate amount to support BSF development [53] on aged manure or manure previously colonized by HFs.

Recent interest in mass-rearing BSFs for food and feed production calls for further investigation of the competitive interactions between HFs and BSFs. These species are ecologically similar in regard to larval diet [54], which can prompt HF infestations in such facilities. This study showed that the presence of HFs may cause a reduction in BSF weight, development time, and survivorship, which in turn, may impact bioconversion and production efficiency. Within a mass production facility, there are few pest control methods that are available, but employing pesticides is not an option as they may kill or contaminate the insects that are intended for food and feed. Therefore, it is important to understand the mechanisms that govern HF control by BSFs in order to avoid or reduce HF infestations.

## 5. Conclusions

This study is the first to evaluate the impact of colonization sequence on development time, survivorship, and weight of HFs and BSFs. Results demonstrate that resource age negatively impacts HF and BSF development and survivorship; however, there appears to be a greater impact on BSFs as those fed manure aged for six or eight days did not complete development to the pre-pupal stage; whereas HFs developed to puparium in all treatments except when they were introduced eight days after BSFs. Findings from this study also reveal that in mixed treatments, HF development and survivorship are not significantly impacted if they colonize poultry manure first, or within two days after the initial introduction of BSFs. Conversely, BSF development and survivorship were negatively impacted when HFs were introduced first or within four days after BSFs. However, poor performance of BSFs in single and mixed treatments may be a result of reduced moisture and nutrients content in aged manure, as well as the amount of resource provided, as previously discussed. Different results may be obtained if this study was performed with more resource. Thus, future studies should investigate this interaction when BSFs are fed optimal amounts of manure.

## References

[1] Begon M., Townsend C.R., Harper J.L. (2006). Ecology: From Individuals to Ecosystems.

[2] Taylor D.B., Moon R.D., Mark D.R. (2012). Economic impact of stable flies (Diptera: Muscidae) on dairy and beef cattle production. J. Med. Entomol..

[3] Larrain P.S., Salas C.F. (2008). House fly (*Musca domestica* L.) (Diptera: Muscidae) development in different types of manure. Chil. J. Agric. Res..

[4] Miranda C.D., Cammack J.A., Tomberlin J.K. (2019). Life-History traits of the house fly, *Musca domestica* L. (Diptera: Muscidae), reared on three manure types. J. Insects Food Feed.

[5] Graczyk T.K., Knight R., Gilman R.H., Cranfield M.R. (2001). The role of non-biting flies in the epidemiology of human infectious diseases. Microbes Infect..

[6] Thomas G.D., Skoda S.R. (1993). Rural Flies in the Urban Environment.

[7] Sawicki R., Lord K. (1970). Some properties of a mechanism delaying penetration of insecticides into houseflies. J. Pestic. Sci..

[8] Afifi S.E., Knutson H. (1956). Reproductive potential, longevity, and weight of house flies which survived one insecticidal treatment. J. Econ. Entomol..

[9] Georghiou G. (1966). Distribution of insecticide-resistant house flies on neighboring farms. J. Econ. Entomol..

[10] Busvine J.R. (1959). Patterns of insecticide resistance to organo-phosphorus compounds in strains of houseflies from various sources. Entomol. Exp. Appl..

[11] El Basheir S. (1967). Causes of resistence to DDT in diazinon-selected and DDT-selected strain of house flies. Entomol. Exp. Appl..

[12] Georghiou G.P., Hawley M.K. (1971). Insecticide resistance resulting from sequential selection of houseflies in the field by organophosphorus compounds. Bull. World Health Organ..

[13] Axtell R.C. (1999). Poultry integrated pest management: Status and future. Integr. Pest Manag. Rev..

[14] Axtell R., Edwards T. (1970). *Hermetia illucens* control in poultry manure by larviciding. J. Econ. Entomol..

[15] Nguyen T.T.X., Tomberlin J.K., Vanlaerhoven S. (2013). Influence of resources on *Hermetia illucens* (Diptera: Stratiomyidae) larval development. J. Med. Entomol..

[16] Sheppard D.C., Newton G.L., Thompson S.A., Savage S. (1994). A value-added manure management-system using the black soldier fly. Bioresour. Technol..

[17] Sheppard C. (1983). Housefly and lesser fly control utilizing the black soldier fly in manure management systems for caged laying hens. Environ. Entomol..

[18] Myers H.M., Tomberlin J.K., Lambert B.D., Kattes D. (2008). Development of black soldier fly (Diptera: Stratiomyidae) larvae fed dairy manure. Environ. Entomol..

[19] Beskin K.V., Holcomb C.D., Cammack J.A., Crippen T.L., Knap A.H., Sweet S.T., Tomberlin J.K. (2018). Larval digestion of different manure types by the black soldier fly (Diptera: Stratiomyidae) impacts associated volatile emissions. Waste Manag..

[20] Furman D.P., Young R.D., Catts E.P. (1959). *Hermetia illucens* (Linnaeus) as a factor in the natural control of *Musca domestica* Linnaeus. J. Econ. Entomol..

[21] Bradley S.W., Sheppard D.C. (1984). Housefly oviposition inhibition by larvae of *Hermetia illucens*, the black soldier fly. J. Chem. Ecol..

[22] Kilpatrick J.W., Schoof H.F. (1959). Interrelationship of water and *Hermetia illucens* breeding to *Musca domestica* production in human excrement. Am. J. Trop. Med. Hyg..

[23] Erickson M.C., Islam M., Sheppard C., Liao J., Doyle M.P. (2004). Reduction of *Escherichia coli* O157:H7 and *Salmonella enterica* serovar enteritidis in chicken manure by larvae of the black soldier fly. J. Food Prot..

[24] Liu Q., Tomberlin J.K., Brady J.A., Sanford M.R., Yu Z. (2008). Black soldier fly (Diptera: Stratiomyidae) larvae reduce *Escherichia coli* in dairy manure. Environ. Entomol..

[25] Schmidtmann E., Martin P. (1992). Relationship between selected bacteria and the growth of immature house flies, *Musca domestica*, in an axenic test system. J. Med. Entomol..

[26] Rochon K. (2003). Persistence and Significance of E. coli in House Flies (Musca Domestica) and Stable Flies (Stomoxys Calcitrans).

[27] Miranda C.D., Cammack J.A., Tomberlin J.K. (2019). Life-History traits of the black soldier fly, *Hermetia illucens* (L.) (Diptera: Stratiomyidae), reared on three manure types. Animals.

[28] Sheppard D.C., Tomberlin J.K., Joyce J.A., Kiser B.C., Sumner S.M. (2002). Rearing methods for the black soldier fly (Diptera: Stratiomyidae). J. Med. Entomol..

[29] Eaton A., Clesceri L., Rice E., Greenberg A., Franson M. (2005). Total suspended solids dried at 103–105 C. Standard Methods for the Examination of Water and Wastewater.

[30] Hogsette J.A. (1992). New diets for production of house flies and stable flies (Diptera, Muscidae) in the laboratory. J. Econ. Entomol..

[31] El Boushy A. (1991). House-fly pupae as poultry manure converters for animal feed: A review. Bioresour. Technol..

[32] Fraenkel G., Bhaskaran G. (1973). Pupariation and pupation in cyclorrhaphous flies (Diptera): Terminology and interpretation. Ann. Entomol. Soc. Am..

[33] Martín-Vega D., Hall M.J., Simonsen T.J. (2016). Resolving confusion in the use of concepts and terminology in intrapuparial development studies of cyclorrhaphous Diptera. J. Med. Entomol..

[34] May B. (1961). The occurrence in New Zealand and the life-history of the soldier fly *Hermetia illucens* (L.)(Diptera: Stratiomyidae). N. Z. J. Sci..

[35] Gligorescu A., Toft S., Hauggaard-Nielsen H., Axelsen J.A., Nielsen S.A. (2019). Development, growth and metabolic rate of *Hermetia illucens* larvae. J. Appl. Entomol..

[36] Brundage A., Benbow M.E., Tomberlin J.K. (2014). Priority effects on the life-history traits of two carrion blow fly (Diptera, Calliphoridae) species. Ecol. Entomol..

[37] Blaustein L., Margalit J. (1996). Priority effects in temporary pools: Nature and outcome of mosquito larva-toad tadpole interactions depend on order of entrance. J. Anim. Ecol..

[38] Weslien J., Djupström L.B., Schroeder M., Widenfalk O. (2011). Long-term priority effects among insects and fungi colonizing decaying wood. J. Anim. Ecol..

[39] Palmer T.M., Young T.P., Stanton M.L. (2002). Burning bridges: Priority effects and the persistence of a competitively subordinate acacia-ant in Laikipia, Kenya. Oecologia.

[40] Schremmer F. (1984). The polymetabol development of the soldier fly larva *Hermetia illucens*—A contribution to the Metamorphosis of the Stratiomyidae. Ann. Nat. Mus. Wien. Ser. B Bot. Zool..

[41] Fatchurochim S., Geden C., Axtell R. (1989). Filth fly (Diptera) oviposition and larval development in poultry manure of various moisture levels. J. Entomol. Sci..

[42] Cammack J.A., Tomberlin J.K. (2017). The impact of diet protein and carbohydrate on select life-history traits of the black soldier fly *Hermetia illucens* (L.)(Diptera: Stratiomyidae). Insects.

[43] Maeda K., Hanajima D., Toyoda S., Yoshida N., Morioka R., Osada T. (2011). Microbiology of nitrogen cycle in animal manure compost. Microb. Biotechnol..

[44] Lalander C.H., Fidjeland J., Diener S., Eriksson S., Vinnerås B. (2015). High waste-to-biomass conversion and efficient *Salmonella* spp. reduction using black soldier fly for waste recycling. Agron. Sustain. Dev..

[45] Ong S.-Q., Lee B.-B., Tan G.-P., Saravanan A., Maniam L. (2017). Capacity of black soldier fly and house fly larvae in treating the wasted rice in Malaysia. Malays. J. Sustain. Agric..

[46] Kim W., Bae S., Park K., Lee S., Choi Y., Han S., Koh Y. (2011). Biochemical characterization of digestive enzymes in the black soldier fly, *Hermetia illucens* (Diptera: Stratiomyidae). J. Asia Pac. Entomol..

[47] Morrison P.E., Davies D.M. (1964). Feeding of dry chemically defined diets + egg production in adult house-fly. Nature.

[48] Bertinetti C., Samayoa A.C., Hwang S.-Y. (2019). Effects of feeding adults of *Hermetia illucens* (Diptera: Stratiomyidae) on longevity, oviposition, and egg hatchability: Insights into optimizing egg production. J. Insect Sci..

[49] Bruno D., Bonelli M., Cadamuro A.G., Reguzzoni M., Grimaldi A., Casartelli M., Tettamanti G. (2019). The digestive system of the adult *Hermetia illucens* (Diptera: Stratiomyidae): Morphological features and functional properties. Cell Tissue Res..

[50] Gobbi P., Martínez-Sánchez A., Rojo S. (2013). The effects of larval diet on adult life-history traits of the black soldier fly, *Hermetia illucens* (Diptera: Stratiomyidae). Eur. J. Entomol..

[51] Jiang C.L., Jin W.Z., Tao X.H., Zhang Q., Zhu J., Feng S.Y., Xu X.H., Li H.Y., Wang Z.H., Zhang Z.J. (2019). Black soldier fly larvae (*Hermetia illucens*) strengthen the metabolic function of food waste biodegradation by gut microbiome. Microb. Biotechnol..

[52] Park S.I., Chang B.S., Yoe S.M. (2014). Detection of antimicrobial substances from larvae of the black soldier fly, *Hermetia illucens* (Diptera: Stratiomyidae). Entomol. Res..

[53] Diener S., Zurbruegg C., Tockner K. (2009). Conversion of organic material by black soldier fly larvae: Establishing optimal feeding rates. Waste Manag. Res..

[54] Čičková H., Newton G.L., Lacy R.C., Kozanek M. (2015). The use of fly larvae for organic waste treatment. Waste Manag..

